# Frequency and Clinicopathologic Characteristics of Triple-Negative Breast Cancer Among Breast Cancer Patients Presenting to Medical Oncology Department, Hayatabad Medical Complex Peshawar, Pakistan

**DOI:** 10.7759/cureus.34581

**Published:** 2023-02-03

**Authors:** Shah Hussain, Fakeeda Durrani, Asif Khan

**Affiliations:** 1 Medical Oncology Unit, Hayatabad Medical Complex Peshawar, Peshawar, PAK; 2 Department of Medicine, Khyber Teaching Hospital, Peshawar, PAK

**Keywords:** clinicopathologic grading, oral contraceptive, laterality, breast cancer, triple negative breast cancer, clinicopathologic features

## Abstract

Introduction: Breast cancer is one of the most common cancers affecting females. The outcomes of this study will aid future research and policy suggestions by giving a rapid local burden and clinicopathologic profile of triple-negative breast cancer (TNBC) patients.

Material and Methods: This cross-sectional study was carried out at the Department of Oncology, Hayatabad Medical Complex Peshawar, Pakistan, from April 21, 2022, to October 21, 2022. The sample size was 120 using a 95% confidence level, 18.7% proportion of TNBC frequency in patients with breast cancer, and 7% absolute precision. All patients who presented with newly diagnosed breast cancer and were between the ages of 30-60 years were included in the study. The study excluded male patients and patients with a history of surgical intervention on the breast during the previous six months.

Results: A total of 120 patients were evaluated. Age ranged between 30-60 years with a mean age of 45 years. Thirty-four (28%) patients were in the age range of 30-45 years and 86 (72%) patients were in the age range of 46-60 years. Fifty-six (47%) patients had BMI ≤27 kg/m^2^ while 64 (53%) had BMI >27 kg/m^2^. The use of oral contraceptives was noted in 25 (21%) patients. A total of 62 (52%) patients had breast cancer on the right side while 58 (48%) had it on the left side.

Conclusion: According to the results of our study, 14% of breast cancer patients had triple-negative disease.

## Introduction

In 2020, there were about 2.3 million new cases of female breast cancer worldwide, accounting for nearly one in four cancer cases amongst women; 685,000 individuals died from it [1]. Since the advent of mammography screening, the incidence of breast cancer has risen and is still rising due to population aging [2]. Some of the most important risk factors include genetic predisposition, estrogen exposure (endogenous and exogenous including long-term hormone replacement therapy (HRT)), radiation exposure, low parity, high breast density, and a history of atypical hyperplasia [3]. Alcohol consumption, obesity, and the Western diet all contribute to the rise in breast cancer cases [4]. There is a noticeable age gradient with 5% of breast cancer cases occurring before age 35 and around 25% before 50 years of age. In Europe, there were 1,814,572 cases of breast cancer estimated to have been diagnosed over the previous five years, and the patients still alive with or without the disease and there were an astounding 6,875,099 cases globally [5]. Because of rising incidence and better treatment results, prevalence is rising [6].

Several variables affect the patient's prognosis and reaction to treatment. The histological type, size, tumor necrosis, lymphovascular invasion, skin, nipple, and chest wall invasion, estrogen receptor (ER) status, progesterone receptor (PR) receptor status, human epidermal growth factor receptor 2 (HER2), cell proliferation marker (ki-67), and type of therapy are the most crucial factors in determining prognosis [7].

The presence of estrogen and progesterone receptors is now the best indicator of prognosis for breast cancer [8]. Intercellular steroid hormone receptors ER and PR have frequently been found in breast cancer. Invasive ductal carcinomas have been observed to have ER expression in 50-80% of cases and PgR expression in 60-70% of cases [9]. Cancers that express ER and PR respond well to hormone treatment and chemotherapy, improving prognosis, boosting survival, and reducing mortality. According to studies from Pakistan and India, there are more hormone receptor-negative cancers than in the population of the west [10].

The most promising breast cancer biomarker aside from hormone receptors is HER2. This protein has prognostic and predictive value and is overexpressed in 18-20% of breast tumors [11]. The prognosis is poorer for tumors that express HER2 and are histologically high grade, prone to spread and express HER2. HER2 overexpression, however, also foretells a favorable response to anti-HER2 medications like trastuzumab and lapatinib.

The current study's goal is to determine the prevalence and clinicopathologic grading of triple-negative breast cancer (TNBC) in breast cancer patients. Breast cancer is not uncommon in our society and early detection and treatment are crucial to lowering morbidity and mortality. Additionally as was already said the degree of a receptor's positivity or negativity directly affects the prognosis and course of treatment for breast cancer and their prevalence differs from community to population. This study's findings will aid future research and policy suggestions by giving us a rapid local burden and clinicopathologic profile of TNBC patients.

## Materials and methods

This cross-sectional study was carried out at the Department of Oncology, Hayatabad Medical Complex Peshawar, Peshawar, Pakistan, from 21/4/2022 to 21/10/2022. Hayatabad Medical Complex is a 1400-bedded tertiary care hospital recognized for undergraduate and postgraduate training. Medical Oncology is the only accredited unit functioning under Khyber Pakhtunkhwa Health Department. This unit has posts of Professor, Assistant Professor, Senior Registrar, Junior Registrar, Medical Officers, Trainee Medical Officers, and trained Staff Nurses. The unit manages a large number of patients with solid tumors and hematological malignancies. Patients come to the unit from all over Khyber Pakhtunkhwa, Federally Administered Tribal Areas (FATA), and Afghanistan. Over 1000 in-patients are taken care of in the unit each year while over 5000 patients are seen in the outpatient department (OPD).

The sample size was 120 using a 95% confidence level, 18.7% proportion of TNBC frequency in patients with breast cancer, and 7% absolute precision. All patients who presented with newly diagnosed breast cancer and were between the ages of 30-60 years were included in the study. The study excluded male patients and those who had a history of surgical intervention on the breast during the previous six months.

After receiving approval from the hospital's ethical and research committee, the study was carried out. Every patient underwent a thorough clinical examination and history. All patients had a biopsy done and the sample was then sent to the hospital laboratory for immunohistochemistry to check for the presence of the ER, PR, and HER2 receptors as well as TNBC. The Nottingham Histologic Score system was used to further evaluate the histological grade and pathological stage of all TNBC patients (TNM scoring system).

A professional histopathologist with a minimum of five years of expertise supervised all laboratory studies. IBM SPSS Statistics for Windows, Version 23.0 (Released 2015; IBM Corp., Armonk, Washington, United States) was used to evaluate the data. Using a Chi-square test with a p-value of 0.05 as significant, TNBC, histologic grade, and pathologic stage were stratified by age, BMI, laterality, and history of oral contraceptive use to examine the effects of the adjustments.

## Results

A total of 120 patients were evaluated. Age ranged between 30-60 years with a mean age of 45 years. Age distribution was analyzed as 34 (28%) patients were in the age range of 30-45 years and 86 (72%) patients were in the age range of 46-60 years. The frequency of TNBC was noted in 17 (14%) patients while 103 (86%) patients didn’t have TNBC (Table 1).

**Table 1:** Stratification of TNBC and non-TNBC with respect to age TNBC: triple-negative breast cancer

	30-45 years	46-60 years	Total	p-value
TNBC, n (%)	6 (18%)	11 (13%)	17 (14%)	0.492
Non-TNBC, n (%)	28 (82%)	75 (87%)	103 (86%)	
Total, n (%)	34 (100%)	86 (100%)	120 (100%)	

Molecular subtypes of breast carcinoma included Luminal A in 58 (56.3%) patients, Luminal B in 26 (25.2%) patients, and HER2 Enriched in 19 (18.4%) patients (Table 2).

**Table 2:** Molecular subtypes of carcinoma breast HER2: human epidermal growth factor receptor 2

Molecular Subtypes	Frequency	Percentage
Luminal A	58	56.3%
Luminal B	26	25.2%
HER2 Enriched	19	18.4%

Fifty-six (47%) patients had BMI ≤27 kg/m^2^ while 64 (53%) patients had BMI >27 kg/m^2^ (Table 3).

**Table 3:** Stratification of TNBC and non-TNBC with respect to BMI status TNBC: triple-negative breast cancer

	BMI ≤ 27 kg/m^2^	BMI > 27 kg/m^2^	Total	p-value
TNBC, n (%)	8 (14%)	9 (14%)	17 (14%)	0.972
Non-TNBC, n (%)	48 (86%)	55 (86%)	103 (86%)	
Total, n (%)	56 (100%)	64 (100%)	120 (100%)	

Positive oral contraceptive use was noted in 25 (21%) patients while 95 (79%) patients had a negative history of use of oral contraceptives (Table 4).

**Table 4:** Stratification of TNBC and non-TNBC with respect to history of oral contraceptives TNBC: triple-negative breast cancer

	Positive History of Oral Contraceptive Use	Negative History of Oral Contraceptive Use	Total	p-value
TNBC, n (%)	5 (20%)	12 (13%)	17 (14%)	0.347
Non-TNBC, n (%)	20 (80%)	83 (87%)	103 (86%)	
Total, n (%)	25 (100%)	95 (100%)	120 (100%)	

Out of 120 patients, 62 (52%) had breast cancer on the right side while 58 (48%) patients had it on the left side (Table 5).

**Table 5:** Stratification of TNBC and non-TNBC with respect to laterality TNBC: triple-negative breast cancer

	Right	Left	Total	p-value
TNBC, n (%)	9 (15%)	8 (14%)	17 (14%)	0.9096
Non-TNBC, n (%)	53 (85%)	50 (86%)	103 (86%)	
Total, n (%)	62 (100%)	58 (100%)	120 (100%)	

The status of histologic grade was analyzed as: eight (47%) patients had histologic grade I, seven (41%) had histologic grade II, and two (12%) patients had histologic grade III (Table 6).

**Table 6:** Stratification with respect to histological grade TNBC: triple-negative breast cancer

Grade	TNBC	Non-TNBC	Total	p-value
Grade I, n (%)	4 (67%)	4 (36%)	8 (47%)	0.370
Grade II, n (%)	2 (33%)	5 (46%)	7 (41%)	
Grade III, n (%)	0 (0%)	2 (18%)	2 (12%)	
Total, n (%)	6 (100%)	11 (100%)	17 (100%)	

## Discussion

According to our study, of the 120 patients, 34 (28%) were between the ages of 30 and 45 years, and 86 (72%) were between the ages of 46-60. A total of 64 (53%) patients had a BMI of ≥27 kg/m^2^. More than 17 (14%) patients were diagnosed with TNBC, of whom eight (47%) had histologic grade I, seven (41%) had histologic grade II, and two (12%) had histologic grade III. 

In their study, Shehzad et al. found that 46 (18.7%) cases were TNBC, which is similar to our findings. Women under the age of 50 made up 71% of these cases [12]. Right breast cancer affected 24 (52.1%) patients, while left breast cancer affected 18 cases (39.1%). There were 41 (89.1%) cases of invasive ductal carcinoma. According to pathological staging, 20 (43.5%) were grade 2, and 26 (56.5%) were grade 3. 33.35% were pT2, 16.66% were pT3, and 50% were pT4.

Hashmi et al., in their study, noted that 205 cases (18.6%) were either non-amplified on HER2/neu gene amplification by fluorescence in situ hybridization (FISH) testing or triple-negative for ER, PR, and Her2/neu [13]. Out of these 205 cases, 110 patients had either radical or conservative primary surgery, and 95 patients had a Tru-Cut biopsy followed by neoadjuvant therapy. The patients were 48.4 years old on average (12.3). Sixty percent of instances were diagnosed when the patient was under 50 years old and only 12.7% of instances were in the pT1 stage, whereas the majority were in the pT2 or pT3 stages. Despite the fact that ductal carcinoma was the most common histologic type, a sizable proportion of cases (10.7% and 5.9%, respectively) displayed metaplastic and medullary-like characteristics. Similarly, significant lymphocyte infiltration and geographic necrosis involving more than 40% of the tumor were notable findings. In 48.2% and 27.3% of patients, respectively, lymph node metastases and lymphovascular invasion were seen.

According to a study by Pediconi et al., TNBC is distinguished from other types of breast cancer by the absence of HER2 expression or amplification as well as the ER and PR receptors [14]. These aggressive, more likely-to-recur-and-metastasize, triple-negative subtypes have drawn a lot of attention recently.

TNBC makes up between 1 -24% of all cases of breast cancer and it mainly affects younger patients and those who have the breast cancer gene 1 (*BRCA1*) mutation. Both morphologically and molecularly, TNBCs exhibit significant heterogeneity; nevertheless, they also share common features like low tumor grade and quick tumor growth. Apocrine, invasive ductal, metaplastic, medullary, and other morphological manifestations of TNBC are possible. Most frequently, a basal phenotype is related molecularly with a special subgroup of malignancies that are not of the basal type and belong to the claudin-low or molecular-apocrine type. Loss of *BRCA1* is frequently correlated with the basal phenotype.

Additionally, Gharekhanloo et al. noted that 190 (10%) patients had TNBC in their study [15]. Both TNBC and non-TNBC had median ages of 59. In comparison to patients without TNBC, there was a considerably larger percentage of African American and Asian patients with TNBC (p = 0.0003). TNBC was substantially correlated with *BRCA1* and *BRCA2* (p = 0.0001, p = 0.0007, respectively). Breast cancer in the past had a strong association with TNBC (p = 0.0003). TNBC and patients with histories of chemoprevention, atypical hyperplasia, or lobular carcinoma in situ (LCIS) were not associated with one another.

According to a local study by Zubair et al., Asians in Pakistan have the second-highest prevalence of breast cancer after Jews in Israel [16]. It is also 2.5 times more common than in nearby Iran and India, where it accounts for 34.6% of female cancers. Although research efforts to date have revealed consanguinity as a significant risk factor for frequent mutations leading to breast cancer and have also shed light on genetic origins in various ethnic groups within Pakistan, the population of Pakistan is still unaware of the epidemiology and etiology of breast cancer.

Despite these findings, 75% of the familial risk of breast cancer remains unknown, highlighting the fact that the majority of breast cancer susceptibility genes are still unknown [17,18]. Global research efforts on various ethnic groups have improved our understanding of the genetic predisposition to breast cancer. The Pakistani population provides a strong genetic pool for shedding light on the genetic etiology of breast cancer [13] because of cousin marriages.

Limitations

The main limitation of our study is the small sample size and being a single-center study. More population-based data from multiple cancer registries addressing the clinical and biological relevance, as well as more epidemiological studies of the various subtypes of breast cancer that incorporate aetiological and lifestyle variables for avoiding incidence and death, are direly needed.

## Conclusions

High-grade breast cancers with a higher frequency of atypical medullary and metaplastic histologies are known as TNBC. In our study, the frequency of TNBC was 14%, and it typically affects younger females under the age of 50.
